# Isolation of *Arcobacter* species and other neglected opportunistic agents from aborted bovine and caprine fetuses

**DOI:** 10.1186/s12917-019-2009-3

**Published:** 2019-07-24

**Authors:** Alessia Di Blasio, Amaranta Traversa, Federica Giacometti, Francesco Chiesa, Silvia Piva, Lucia Decastelli, Alessandro Dondo, Silvia Gallina, Simona Zoppi

**Affiliations:** 1 Istituto Zooprofilattico Sperimentale del Piemonte, Liguria e Valle d’Aosta, Via Bologna 148, 10154 Torino, Italy; 2 Department of Veterinary Medical Sciences, University of Bologna, Via Tolara di Sopra 50, 40064, Ozzano Emilia, Bologna, Italy; 3 Department of Veterinary Medical Sciences, University of Torino, Largo Paolo Braccini 2, Grugliasco, 10095 Torino, Italy

**Keywords:** *Arcobacter* species, Opportunistic emerging pathogens, Aborted fetuses, Co-infections

## Abstract

**Background:**

Infectious abortion in ruminants is a problem in animal husbandry worldwide. It is important to obtain a diagnosis, to make sure that proper control measures can be instituted, but most abortion cases remain without an etiologic diagnosis. This report describes the presence of *Arcobacter* species and several neglected opportunistic abortifacient agents in ruminant abortion cases showing or not co-infections among at least one of the major recognized protozoal, fungal, bacterial and viral abortifacient agents.

**Results:**

A total of 67 fetuses (55 cattle and 12 goats) and just one placenta (cattle) were considered. Among the most common abortive agents, *Neospora caninum* (19,4%), followed by *Chlamydophila abortus* (4,5%), *Listeria monocytogenes* 1/2a (2,98%), *Bovine Viral Diarrhea Virus* type 1b (2,98%), *Bovine herpesvirus 4* (2,98%), and *Aspergillus* spp. (2,98%) were detected. The isolated neglected opportunistic bacteria include *Escherichia coli*, *Acinetobacter lwoffii*, *Staphylococcus* spp., *Streptococcus* spp., *Streptococcus uberis*, *Streptococcus suis*, *Trueperella pyogenes, Mannheimia haemolytica*, *Bacillus cereus* and *Nocardia* spp. Other bacterial species, not associated with abortion by literature, but described as causes of diseases occurring sporadically both in humans and animals, were also detected. Three *Arcobacter* strains, namely two *A. skirrowii* and one *A. cryaerophilus*, were isolated from 3 bovine aborted fetuses, and *A. butzleri* was isolated from the placenta.

**Conclusions:**

A not negligible isolation of *Arcobacter* species and other neglected abortifacient agents has to be mentioned, with prevalences that seem to be emerging and replacing or co-placing the major infectious players in bovine and caprine reproductive failure due to abortion disease, even if further studies investigating the aetiological power and transmission routes are needed in order to define the role of these microrganisms in ruminant abortion.

## Background

Infectious abortion in ruminants is a problem in animal husbandry worldwide, and abortion in cattle, with an estimated rate from 2 to 5%, is widely acknowledged to cause considerable economic losses and, despite extensive histopathologic, microbiologic, and molecular investigations, most bovine abortion cases remain without an etiologic diagnosis [1]. The major infectious agents involved in ruminant abortion are *Neospora caninum, Bovine pestiviruses, Bovine herpesvirus* 1 and 4, *Parainfluenza virus* type 3, *Brucella* spp., *Chlamydophila abortus, Coxiella burnetii, Campylobacter fetus, Salmonella* sp., *Listeria* spp., and *Aspergillus* spp.. However, most bacterial causes of abortion are opportunistic pathogens. These organisms are not infectious, and are common inhabitants of the host or its environment and might include *Arcanobacterium pyogenes* and *Bacillus* spp. followed by *Escherichia coli*, *Histophilus somni*, *Pasteurella* spp., *Staphylococcus* spp., *Streptococcus* spp., and any other bacteria that can find their way into the bloodstream can cause sporadic abortion [2]. Moreover *Arcobacter* species have been reported to be involved in animals abortion [3, 4].

The first report of bacteria now known as arcobacters was originally described by Ellis et al. (1977) [5] as an isolation of “Spirillum/Vibrio-like organisms” from placenta and internal organs of bovine fetuses; these microorganisms were initially assigned to the genus *Vibrio*, but, considering their failure in fermenting carbohydrates, they were subsequently transferred into the genus *Campylobacter* as an atypical and heterogeneous group of aerotolerant campylobacters [6]. In 1991, the genus *Arcobacter* was created by Vandamme and Colleagues to accommodate these two atypical campylobacters, *Campylobacter nitrofigilis* [7] and *Campylobacter cryaerophila* [6] and, in 1992, the genus was enlarged with the reclassification of *Campylobacter butzleri*, isolated from human stools but also from animals [8], as *Arcobacter butzleri*, and with the inclusion of a new species, *Arcobacter skirrowii*, isolated predominantly from preputial fluids of bulls or from bovine, porcine, and ovine aborted fetuses, as well as from diarrhoeic feces [9]. Hitherto, a total of 27 *Arcobacter* species have been described in a wide variety of ecological niches and from various hosts and environments, mainly described from water-related environments and shellfish [10]. Arcobacters are considered emerging bacterial zoonotic agents: *A. butzleri*, *A. cryaerophilus* and *A. skirrowii* are the species most commonly described in clinical conditions, namely associated with human and animal disease. Furthermore, as the *Arcobacter* organisms are commonly isolated from fecal samples of healthy animals and humans, and they are unable to satisfy the Koch’s postulate, their pathology and pathogenicity are under dispute [11]. In animals, arcobacters may play a role in animal reproductive disorders, such as infertility and late-term abortions in cattle, pigs, sheep [12], equine and alpaca [13]. In literature, the species involved in abortions are *A. cryaerophilus*, *A. skirrowii*, *A. butzleri*, *A. thereius* [3] and *A. porcinus* [4]. However, since the first isolation, arcobacters were reported in bovine abortions only in three cases [14–16] and, differently from pigs, no studies in literature have clearly identified the role of the genus *Arcobacter* as abortifacient pathogen and as causative agent of disease in cattle.

This report is to describe the isolation of *Arcobacter* species, long time since the last description in literature, and other neglected opportunistic abortifacient agents from the abomasum’s content of aborted fetuses both of cattle and small ruminants coming from a passive Bovine Brucellosis (BB) Surveillance National plan performed in Piedmont, an officially BB-free Italian region. The aim of this study is presenting important and inspiring data on the isolation of *Arcobacter* species and other bacterial agents that seem to be emerging and replacing or co-placing the major infectious players in bovine reproductive failure due to abortion disease.

## Results

A total of 67 fetuses (*n* = 55 cattle and *n* = 12 goats) and just one placenta (cattle) were collected and analyzed. The absence of gross lesions at necropsy was reported in almost all (*n* = 64) the fetuses. The findings of the diagnostic approach showed that: i) 25 fetuses (37,3%) were negative for any protozoal, fungal, bacterial and viral agents investigated; ii) 22 fetuses (32,8%), 19 bovine and 3 caprine, were positive only to routine bacteriological exam on blood agar plates (Table 1); iii) 10 fetuses (14,9%), 8 bovine and 2 caprine, showed a single positivity for only one among the investigated pathogens, namely *N. caninum* (n = 6), *C. abortus* (*n* = 2)*, L. monocytogenes* 1/2a (*n* = 1) and *Bovine Viral Diarrhea Virus* (BVDV) type 1b (n = 1); iv) 10 fetuses (14,9%) showed different coinfections among recognized protozoal, fungal, bacterial and viral abortifacient agents, and, three *Arcobacter* strains, two *A. skirrowii* and one *A. cryaerophilus*, were isolated from 3 among these 10 bovine aborted fetuses (Table 2). Moreover, a strain of *A. butzleri* was isolated from the placenta, in purity. Among the overall investigated *Arcobacter* virulence genes, only *cia*B and *mvi*N were detected and were present in all the isolates.

**Table 1:** Positive bacteriological findings of the routine exam on blood agar for abortion cases

Animal species	Necropsy	Bacteriology
Caprine	No Visible Lesion	*Staphylococcus equorum, Aerococcus viridans*
Caprine	No Visible Lesion	*Staphylococcus equorum, Aerococcus viridans*
Caprine	No Visible Lesion	*Staphylococcus equorum*
Bovine	Congenital goiter and heart defect	*Acinetobacter lwoffii, Staphylococcus sciuri, Staphylococcus lentus, Escherichia coli*
Bovine	Gelatinous subcutaneous oedema, hepatomegaly	*Trueperella pyogenes*
Bovine	Generalized oedema	*Escherichia coli, Streptococcus alactolyticus*
Bovine	No Visible Lesion	*Streptococcus suis*
Bovine	No Visible Lesion	*Escherichia coli*
Bovine	No Visible Lesion	*Escherichia coli*
Bovine	No Visible Lesion	*Corynebacterium* spp., *Staphylococcus lentus, Pseudomonas* spp.
Bovine	No Visible Lesion	*Streptococcus uberis*
Bovine	No Visible Lesion	*Bacillus cereus, Burkholderia cepacia*
Bovine	No Visible Lesion	*Escherichia coli*
Bovine	No Visible Lesion	*Pseudomonas* spp.
Bovine	No Visible Lesion	*Aerococcus viridans, weeksella viros/ Bergeyella zoohelcum*
Bovine	No Visible Lesion	*Pantoea ananatis*
Bovine	No Visible Lesion	*Acinetobacter lwoffii, Corynebacterium glutamicum*
Bovine	No Visible Lesion	*Nocardia* spp*., Sphingomonas paucimobilis*
Bovine	No Visible Lesion	*Escherichia coli*
Bovine	No Visible Lesion	*Enteroccus faecium*
Bovine	No Visible Lesion	*Acinetobacter lwoffii, Streptococcus* spp.
Bovine	No Visible Lesion	*Escherichia coli*

Abortion cases with positive bacteriological findings on the routine exam on blood agar with description of the observed anatomopathological lesions, animal species and bacterial species isolated

**Table 2:** Abortion cases showing co-infections among at least one of the major recognized protozoal, fungal, bacterial and viral abortifacient agents

Animal Species	Bacteriology & Micology	*N. caninum*	*C. abortus*	*Arcobacter* spp.	*BOHV4*	*BVDV*	*Listeria* spp.
Bovine	NEG	POS	NEG	NEG	POS	NEG	NEG
Bovine	*Acinetobacter lwoffii, Aerococcus viridans* *Aspergillus* spp.	POS	NEG	*A. skirrowii*	NEG	NEG	NEG
Bovine	NEG	NEG	NEG	*A. cryaerophilus*	NEG	BVD 1b	NEG
Bovine	*Acinetobacter lwoffii, Escherichia coli*	POS	NEG	NEG	NEG	NEG	NEG
Bovine	*Aspergillus* spp.	NEG	NEG	NEG	NEG	NEG	*L. innocua*
Bovine	*Escherichia coli*	POS	NEG	NEG	NEG	NEG	NEG
Bovine	*Mannheimia haemolytica*	POS	NEG	NEG	NEG	NEG	NEG
Bovine	NEG	POS	NEG	NEG	POS	NEG	NEG
Bovine	NEG	POS	NEG	*A. skirrowii*	NEG	NEG	NEG
Caprine	*Staphylococcus epidermidis, Staphylococcus equorum*	NEG	POS	NEG	NEG	NEG	NEG

POS: positive; NEG: negative

## Discussion

Establishing a definitive cause of bovine abortion is a challenging problem faced by veterinary practitioners and diagnosticians [17] and the knowledge of the most common causes of bovine abortion is useful to set up interventions able to mitigate future fetal loss in the herd. A not negligible percentage of fetuses with negative results for infectious abortion agents and the almost overall absence of anatomopathological lesions have to be mentioned in this report, in line with the literature, and highlighting, once again, as these scenarios can interfere with the recovery of causative agents.

Among the 42 fetuses in which an infectious agent was identified, bacterial agents, followed by viral, protozoal and fungal agents were identified. The most common abortive agent detected resulted *N. caninum* (*n* = 13, 19,4%), followed by *C. abortus* (*n* = 3, 4,5%), *L. monocytogenes* 1/2a (*n* = 2, 2,98%), BVDV type 1b (n = 2, 2,98%), *Bovine herpesvirus 4* (n = 2, 2,98%), and *Aspergillus* spp. (n = 2, 2,98%). However, several abortion cases with multiple possible etiologies were detected, both in the 10 cases with co-infections among the well-recognized abortifacient agents (Table 2) as well as in the 22 cases with positive bacterial cultures on blood agar plates that are not primary abortifacient agents (Table 1). In fact, whereas *Neospora* spp., *C. abortus*, *L. monocytogenes*, BVDV and BoHV4 are recognized as abortifacient agents, the other isolated bacterial species are usually considered pathogenic or opportunistic or commensal or environmental bacteria. Recent studies indicate that uterus is not sterile during pregnancy and potentially pathogenic bacteria can be present in the placenta of term pregnancies without causing abortion or inflammation [18], and therefore, the bacterial species not described as primary abortifacient agents could play a role as opportunistic pathogens, components of the uterine microbiota, or contaminants of the post-abortive phase. In our study, several abortion cases contained different bacterial species, recovered from the routine bacteriological exam on blood agar plates, that could be classified as possible abortive bacterial agents, given they were previously reported by literature as opportunistic abortive bacteria, namely *Escherichia coli* (*n* = 9), *Acinetobacter lwoffii* (*n* = 5), *Staphylococcus* spp. (n = 5), *Streptococcus* spp. (*n* = 1), *S. uberis* (n = 1), *S. suis* (n = 1), *Trueperella pyogenes* (n = 1*), Mannheimia haemolytica* (n = 1), *Bacillus cereus* (n = 1) and *Nocardia* spp. (n = 1) [2, 17, 19–25]. On the contrary, other bacterial species isolated in the present study (*Staphylococcus sciuri, Staphylococcus equorum*, *Staphylococcus epidermidis*, *Streptococcus suis, Streptococcus alactolyticus, Corynebacterium* spp., *Burkholderia cepacia, Weeksella viros/Bergeyella zoohelcum*, *Pantoea ananati, Sphingomonas paucimobilis*), have never been associated with abortion even if they have been described as causes of diseases occurring sporadically both in humans and animals [26–36]. As previously suggested by Vidal et al. (2017) mixed infections may play an underestimated role in ruminant abortions. In fact, it is often difficult to assess the presence of potentially infectious bacteria in cultures, particularly in cases where rare opportunistic agents are identified, as they tend to be overlooked as contaminants, but, similarly, complementary pathological analyses are needed to avoid overinterpretation of mere detection of the opportunistic pathogens [22].

Regarding the association of *Arcobacter* spp. with infertility and abortion in livestock animals, it has been reported worldwide but its role in abortions was investigated predominantly in pigs, in which the transmission of *Arcobacter* species was demonstrated from carrying sows to their offspring, both vertically and horizontally [37]. On and Colleagues in 2002 [38], reported, in one hand, a high *Arcobacter* prevalence in Danish pig abortions, in cases in which no established abortifacient agent is detected, but in the other, they recommend that other studies are needed to define the role of each *Arcobacter* species, especially where co-infections with other agents are present, suggesting that some *Arcobacter* strains play a primary role in abortion while others may be opportunistic pathogens colonizing the fetus.

In our report, *Arcobacter* species resulted the most isolated bacteria in bovine fetuses among the 10 cases with co-infections, with *N. caninum* in two cases and BVD type 1b in one other case; in addition, in two of these cases *A. skirrowii* and *A. cryaerophilus* resulted the only bacterial species isolated. To note that *Arcobacter* species were never isolated from blood agar plates performed during routine microbiological exam with incubation temperature of 37 °C; in case of other microbial contamination, *Arcobacter* species are masked by the other populations that overgrow on plates, or the higher incubation temperature could hinder the *Arcobacter* growth, and, therefore, a selective bacteriological approach should be the better choice in order to evaluate the presence of the genus *Arcobacter*. The true incidence and data on *Arcobacter* species on animal abortion are presumed to be underreported and probably greatly underestimated.

This report revived the presence of *Arcobacter* spp. in bovine abortion and described as *A. cryaerophilus* and *A. skirrowii* could have had an active but not exclusive role in the observed fetal deaths and, consequently, in the observed abortion cases. Differently from what observed in sows by On et al. (2002) [38], the isolation of *A. cryaerophilus* and *A. skirrowii* was reported in all the described cases in addition to other organisms frequently associated with bovine abortion (*N. caninum* and BVDV), and therefore, authors supposed an opportunistic role, if there was, or at least superinfection of *Arcobacter* spp.. A synergic effect could have occurred on the pathogenesis of the abortion in which several determinants have been explicated their pathogenicity: on the basis of the recovery of *A. cryaerophilus* and *A. skirrowii* in the abomasum’s content, it is possible to hypothesize an exceeding of the placental barrier, thanks to the injurious or immunosuppressive state induced by other pathogens (*N. caninum* and BVDV), otherwise, since *Arcobacter* species have been isolated from several environments and hosts [39, 40], an ascending infection by vaginal pathway or a post-fetal ascendant invasion by *Arcobacter* spp. could also be hypothesized. The isolation of *A. butzleri* in the only one placenta submitted to the laboratory immediately after its expulsion, has to be described, even if no specific conclusions could be extrapolated. Collection and submission of the placenta should be recommended, if it can be sampled, because it is equally important and could contribute with the entire fetus to abortion diagnosis, but a whole, intact placenta is often rarely received [2], as confirmed by our report.

In our study, no carcasses showed gross lesions, suggesting an indirect action of pathogens on the fetus by impairment of the maternal-fetal circulation (*N. caninum*) or the mother’s immune system failure (BVDV) [2]. The breakable conservation of abortion products leads often to receive specimens in poor condition not suitable for histopathology purposes, and, consequently, a lack of data about the presence and the type of microscopic lesions could limit the definition of the role of each determinant involved in abortion cases.

## Conclusions

Although *N. caninum* resulted the most common agent of bovine abortions, demonstrating a consistent presence of the organism in cattle abortion cases and, therefore confirming in line with literature that it is still a cattle problem, a not negligible isolation of *Arcobacter* species has to be mentioned, with prevalence in line with the major abortifacient agents, as well as the isolation of other opportunistic, commensal or environmental bacteria, even if with lower occurrence levels. The frequent isolation of these bacteria should be taken into account in order to assess if the once-major abortion players could be or are being replaced by an increase in these opportunistic pathogens that are identified as possible abortive agents but that remain in the dark zone of knowledge and that seem to be emerging and needing immediate attention. Further studies are needed in order to both identify these emerging opportunistic bacteria, whose incidence in ruminant abortion is likely underestimated, and investigate the transmission routes and the specific role of *Arcobacter* in bovine abortion.

## Methods

Aborted fetuses coming from a passive BB Surveillance plan in the Piedmont region, in Italy, were included in this report. All abortion products, collected from bovine and caprine herds between October 2017 to July 2018 in the area covered by the Istituto Zooprofilattico Sperimentale del Piemonte, Liguria e Valle d’Aosta (IZSPLV), that includes three Italian regions (Piedmont, Liguria, and the Aosta Valley) of the Northwest Italy, were received at IZSPLV laboratory. Samples and analyses included in the present study were performed in accordance with current Regulation concerning of Maintenance of Bovine Brucellosis free Health Status (Directive 97/12/CE, adopted by Italian Law 196/199). Firstly, the carcasses were submitted to necropsy and then followed the application of the routine diagnostic protocol for the detection of the major infectious agents of abortion, namely *Salmonella* sp., *Listeria* spp., *Brucella* spp., *C. fetus, C. abortus, C. burnetii, N. caninum, Bovine pestiviruses, Bovine herpesvirus* 1 and 4, *Parainfluenza virus* type 3, fungi and yeasts. See Table 3 for more details and references [41–47]. In addition, a routine bacteriological examination on blood agar plates (Columbia agar, Liofilchem s.r.l., Roseto degli Abruzzi, Italy) incubated at 37 °C was performed for each fetus sample considered in the study from the abomasum’s content. This protocol has been extended with a selective bacteriological exam for the isolation of *Arcobacter* spp. [48]. In detail, 1 ml of this specimen was enriched in Preston broth (Microbiol & C. snc, Cagliari, Italy) under Carbon Dioxide-enriched atmosphere condition at 30 °C; every 24 h for the following 3 days, broth culture were subcultured, under the same conditions, on Charcoal Cefoperazone Deoxycholate Agar (CCDA; Oxoid, Milan, Italy) added with CAT supplement, cefoperazone (8 mg l-1), amphotericin (10 mg l-1), teicoplanin (4 mg l-1) (Oxoid, Milan, Italy). CAT-CCDA plates were observed for 5 days and on suspected colonies Gram staining was performed. According to the protocols described by Douidah (2010, 2012) [49, 50], the strains identified morphologically as *Arcobacter* spp. were processed with two end-point multiplex PCR in order to identify the species and to detect virulence genes (*cad*F, *pld*A, *tly*A, *cia*B, *irg*A, *hec*A, *mvi*N, cj1349 and *hec*B). All the considered strains were identified or confirmed using Vitek-MS (bioMerieux Italia, Florence, Italy). The reference strains, *A. butzleri* DSM 8739TM, *A. cryaerophilus* DSM 7289TM, and an *A. skirrowii* field strain, confirmed by Sanger sequencing, were used as positive control.

**Table 3:** Diagnostic protocol applied on the considered aborted fetuses

Pathogen	Matrix	Test and references	Animal species
*Bovine herpesvirus* 1	Lung	FAT^a^, PCR [41]	Cattle
*Bovine herpesvirus* 4	Lung	FAT^a^	Cattle
*Pestiviruses*	Spleen	ELISA^b^, PCR [42]	Cattle; Small Ruminants
*Brucella* spp.	Content of abomasum Pool of organs	Bacteriological [43]	Cattle; Small Ruminants
*Campylobacter fetus*	Content of abomasum Liver	Bacteriological [44], PCR [41]	Cattle; Small Ruminants
*Chlamydophila abortus*	Liver	PCR [41]	Small Ruminants
*Coxiella burnetii*	CNS, Liver, Kidney, Lung	PCR [41]	Cattle; Small Ruminants
Fungi and yeasts	Content of abomasum	Bacteriological [45]	Cattle; Small Ruminants
*Listeria* spp.	Content of abomasum	Bacteriological [46]	Cattle; Small Ruminants
*Neospora caninum*	CNS, Liver, Kidney, Lung	PCR [41]	Cattle
*Parainfluenza virus* type 3	Lung	FAT^a^	Cattle
*Salmonella* sp.	Liver	Bacteriological [47]	Cattle; Small Ruminants

Diagnostic protocol currently applied on the considered aborted fetuses in the IZSPLV laboratory with indication, for each pathogen, of the considered matrix and test used for its isolation and or detection and the considered animal species. CNS: central nervous system; ^a^FAT: fluorescence antibody testing; internal method with polyclonal antiserum conjugated to fluorescein isothiocyanate (Veterinary Medical Research & Development, Pullman, WA, USA). ^b^IDEXX BVDV Ag/Serum Plus, Switzerland

## Data Availability

The datasets generated during the current study are not publicly available. However, the data can be available from Authors upon request (please contact Alessia Di Blasio, Alessia.DiBlasio@izsto.it).

## Abbreviations

BB: Bovine Brucellosis
BVDV: *Bovine Viral Diarrhea Virus*
IZSPLV: Istituto Zooprofilattico Sperimentale del Piemonte, Liguria e Valle d’Aosta
